# RORα2 requires LSD1 to enhance tumor progression in breast cancer

**DOI:** 10.1038/s41598-017-12344-0

**Published:** 2017-09-20

**Authors:** Kyeongkyu Kim, Ji Min Lee, Young Suk Yu, Hyunkyung Kim, Hye Jin Nam, Hyeong-Gon Moon, Dong-Young Noh, Keun Il Kim, Sungsoon Fang, Sung Hee Baek

**Affiliations:** 1 Creative Research Initiatives Center for Chromatin Dynamics, School of Biological Sciences, Seoul National University, Seoul, 151-742 South Korea; 2 Department of Surgery and Cancer Research Institute, Seoul National University College of Medicine, Seoul, 110-744 South Korea; 3 Department of Biological Sciences, Sookmyung Women’s University, Seoul, 140-742 South Korea; 4 Severance Biomedical Science Institute, BK21 PLUS project for Medical Science, Yonsei University College of Medicine, Seoul, South Korea

## Abstract

Retinoic acid-related orphan receptor α (RORα) regulates diverse physiological processes, including inflammatory responses, lipid metabolism, circadian rhythm, and cancer biology. RORα has four different isoforms which have distinct N-terminal domains but share identical DNA binding domain and ligand binding domain in human. However, lack of specific antibody against each RORα isoform makes biochemical studies on each RORα isoform remain unclear. Here, we generate RORα2-specific antibody and characterize the role of RORα2 in promoting tumor progression in breast cancer. RORα2 requires lysine specific demethylase 1 (LSD1/KDM1A) as a coactivator for transcriptional activation of RORα2 target genes, exemplified by *CTNND1*. Intriguingly, RORα2 and LSD1 protein levels are dramatically elevated in human breast cancer specimens compared to normal counterparts. Taken together, our studies indicate that LSD1-mediated RORα2 transcriptional activity is important to promote tumor cell migration in human breast cancer as well as breast cancer cell lines. Therefore, our data establish that suppression of LSD1-mediated RORα2 transcriptional activity may be potent therapeutic strategy to attenuate tumor cell migration in human breast cancer.

## Introduction

RORα, a member of the orphan nuclear receptor family, plays various roles in signal integration including modulation of homeostasis and disease by positively or negatively regulating subsets of gene expression^1^. RORα functions as potent regulators of normal physiology and pathologies such as cancer^2^. RORα binds to hormone response elements composed of a 6-bp A/T-rich sequence preceding a half-site core motif PuGGTCA (RORE) as a monomer or homodimers, and controls its target gene transcription^3^. RORα binding sites are present in numerous gene promoter regions, such as *N-myc, γF-crystallin, caveolin-3, and purkinje cell protein* 2 *and 4* ^4–6^, indicating that RORα is involved in various biological processes.

By alternative RNA splicing, RORα has four different isoforms in human, whereas mice have only two isoforms, RORα1 and 4^3,7,8^. The difference of N-terminal domain (NTD) in each RORα isoform confers different DNA binding specificities as well as transcriptional activities. RORα1 has been shown to reduce accumulation of reactive oxygen species (ROS) by conferring resistance to oxidative stress-induced apoptosis^9^. Expressions of RORα1 and RORα4 are induced by hypoxia or ER stress, suggesting that the functional role of RORα may be positively involved in cellular stress responses^10,11^. Compared to RORα1 and RORα4 which show ubiquitous expression pattern, the expression patterns of RORα2 and RORα3 are tissue- and cell type-specific^12,13^.

Transcriptional regulation requires a functional crosstalk between transcription factors and chromatin-modifying enzymes. Lysine-specific demethylase 1 (LSD1/KDM1A) participates in gene repression process as a part of the REST corepressor (CoREST) complexes mediating the demethylation of H3K4me1/2, but also participates in gene activation process associated with androgen receptor through demethylation of H3K9me1/2^14,15^. LSD1 participates in the progression of many types of cancer. LSD1 is highly expressed in ER-negative breast cancer and prostate carcinomas^16^. In neuroblastoma, LSD1 participates in the maintenance of malignancy^17^. Further, LSD1 suppresses *PTEN* gene expression with an orphan nuclear receptor, TLX^18^. We have reported tumor suppressive function of RORα, demonstrating that RORα attenuates Wnt/β-catenin signaling by PKCα-dependent phosphorylation in colon cancer cells and that RORα enhances p53-dependent apoptotic function to inhibit tumor progression^2,19^. We have also reported the oncogenic role of EZH2 is enhanced by degradation of RORα in methylation-dependent manner, thereby inhibiting the tumor suppressive role exerted by RORα^20^.

Here, we report that RORα2 is critical to promote cell proliferation and migration in human breast cancer cells. RORα2 requires LSD1 as a coactivator for transcriptional activation of target genes. Using specific antibody against RORα2, we show that both RORα2 and LSD1 protein levels are elevated in breast cancer tissue specimens compared to the matched normal tissue specimens. Altogether, our data indicate that RORα2 requires LSD1 to enhance cell migration and tumor progression in human breast cancer.

## Results

### RORα2 associates with LSD1

**Figure 1:**
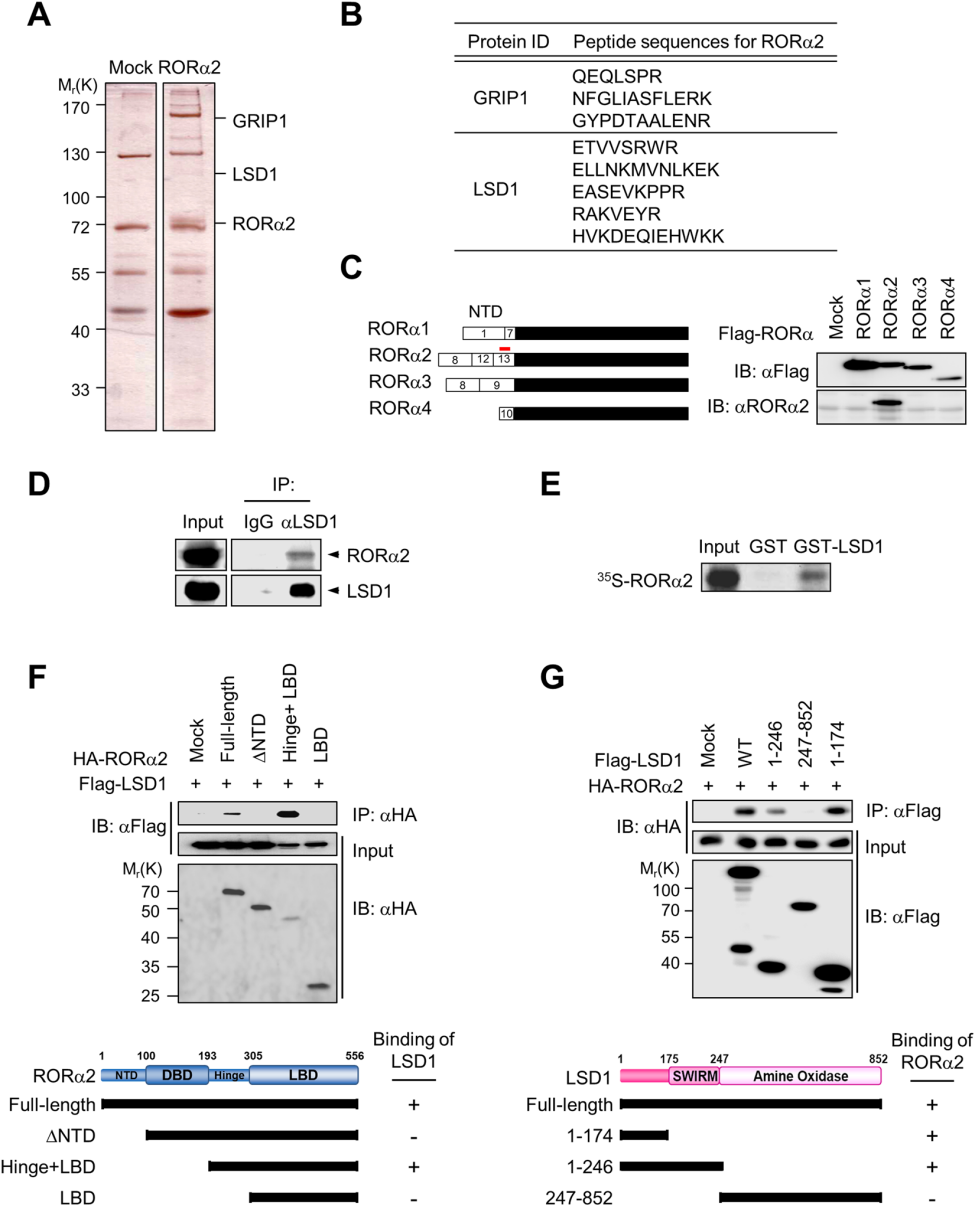
Identification of LSD1 as a binding partner of RORα2. (**A**) RORα2-binding proteins were purified from extracts obtained from HEK293 cells stably expressing Flag-tagged RORα2 by co-immunoprecipitation using anti-Flag antibody. The bound proteins were resolved by SDS-PAGE and prepared for LC-MS/MS analysis. (**B**) Peptide sequences of RORα2-associated polypeptides obtained from LC-MS/MS analysis. (**C**) A schematic diagram of isoforms of the RORα. Numbers in the boxes represent exon numbers that constitute the NTD of RORα1, RORα2, RORα2 and RORα4. Location of antigen that used to generate specific RORα2 antibody is marked with red line (left panel).Validation of RORα2-specific antibody by immunoblot using cell extracts obtained from HEK293T overexpressing mock, RORα1, RORα2, RORα3 and RORα4 (right panel). (**D**) Co-immunoprecipitation of endogenous RORα2 with LSD1 in HEK293T cells. (**E**) GST pull-down assay was conducted using *in vitro*-translated ^35^S-methionine-labeled RORα2 with GST-LSD1 protein. (**F**) Co-immunoprecipitation assay using anti-HA antibody revealed that the hinge domain of RORα2 is sufficient to bind LSD1. Whole-cell extracts and co-immunoprecipitated material with anti-Flag antibody were analyzed by immunoblot against anti-HA or anti-Flag antibody (upper panel). Illustration of the deletion fragments of RORα2 is shown in bottom panel. (**G**) Co-immunoprecipitation assay using anti-Flag antibody revealed that the SWIRM domain of LSD1 is sufficient to bind RORα2. Whole-cell extracts and co-immunoprecipitated material with anti-Flag antibody were analyzed by immunoblot against anti-HA or anti-Flag antibody (upper panel). Illustration of the deletion fragments of LSD1 is shown in bottom panel.

To define the roles of RORα2, we searched for the RORα2-interacting proteins by liquid chromatography mass spectrometry/mass spectrometry (LC-MS/MS). The LC-MS/MS analysis revealed that glucocorticoid receptor-interacting protein 1 (GRIP1)^21^, a well-known binding partner of RORα, was identified as an RORα2-interacting protein (Fig. 1A,B). Intriguingly, histone demethylase LSD1 was identified as an RORα2-interacting molecule (Fig. 1A,B), indicating the possibility of the functional link between RORα2 and LSD1.

Next, we generated a specific antibody against the NTD of RORα2 (Fig. 1C, left panel) and confirmed no cross-reactivity of RORα2-specific antibody with other known RORα isoforms by immunoblot analysis (Fig. 1C, right panel). Using RORα2-specific antibody, we examined if RORα2 associates with LSD1 in HEK293 cells. Cell extracts were immunoprecipitated with antibody against LSD1 or control IgG, and the immunoprecipitates were analyzed by immunoblot. RORα2 was detected from the anti-LSD1 immunoprecipitates, indicating that RORα2 associates with LSD1 at endogenous expression level in HEK293 cells (Fig. 1D). To examine whether LSD1 directly interacts with RORα2, GST fusions with LSD1 that have been bound to glutathione-Sepharose were incubated with ^35^S-labeled RORα2. GST-pull down assay revealed that ^35^S-labeled RORα2 efficiently bound to full length of LSD1 *in vitro* (Fig. 1E).

To determine the binding domain of RORα2 with LSD1, various deletion mutants of RORα2 were co-expressed with Flag-tagged LSD1 in HEK293 cells. Co-immunoprecipitation assay indicated that hinge domain of RORα2 is required for the interaction with LSD1 (Fig. 1F). We further determined the binding domain of LSD1 with RORα2. We observed that the NTD of LSD1 including the SWIRM domain is required for interaction with RORα2, whereas no physical interaction was detected between the C-terminal domain of LSD1 and RORα2 (Fig. 1G). These results indicate that LSD1 is a binding partner of RORα2 and that hinge domain of RORα2 and N-terminal SWIRM domain of LSD1 are sufficient for the interaction between RORα2 and LSD1.

### LSD1 functions as a coactivator for RORα2-dependent transcription

**Figure 2:**
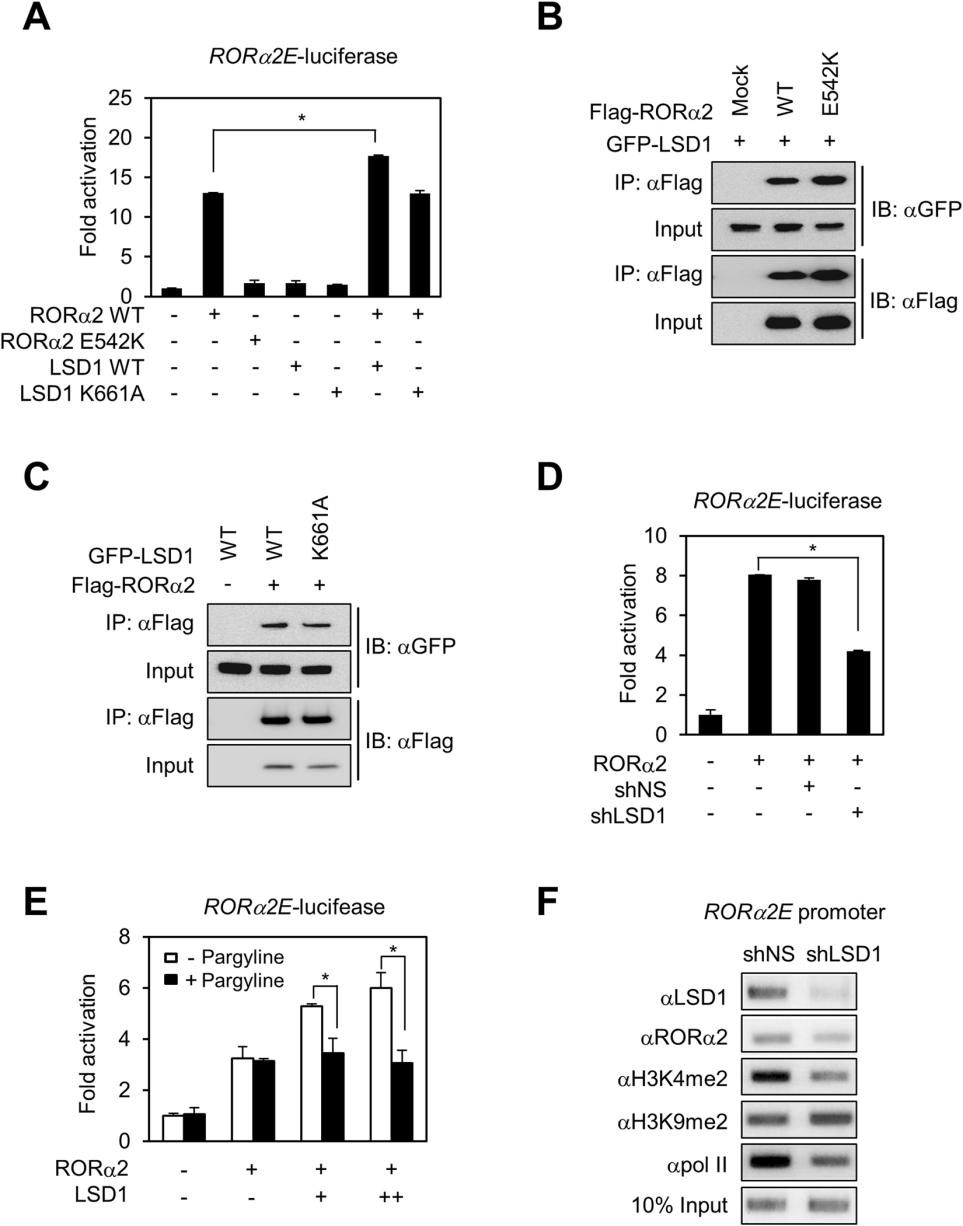
LSD1 increases transcriptional activity of RORα2. (**A**) Transcriptional activation of the *ROR*α*2E*-luciferase reporter by RORα2 WT or E542K mutant with LSD1 WT or K661A mutant in HEK293T cells. Cells were transfected with either 100 ng of *RORa2E* luciferase reporter along with 100 ng of RORα2 WT/RORα2 E542K or 100 ng of LSD1 WT/LSD1 K661A. Results are expressed as fold activation compared to empty vector. Data are represented as mean ± S.E.M. for three independent experiments. *P* value is shown from Student’s *t* test analysis. *p < 0.001. (**B**) Binding affinity of RORα2 WT or E542K mutant with LSD1 was assessed in HEK293 cells expressing indicated constructs. (**C**) Binding affinity of LSD1 WT or K661A mutant with RORα2 was assessed in HEK293 cells expressing indicated constructs. (**D**) Effect of LSD1 knockdown on *ROR*α*2E*-luciferase reporter with overexpression of RORα2 in HEK293T cells. Cells were transfected with either 100 ng of *RORa2E* luciferase reporter along with 100 ng of RORα2 or 400 ng of mock/LSD1 shRNA. Results are expressed as fold activation compared to empty vector. Data are represented as mean ± S.E.M. for three independent experiments. *P* value is shown from Student’s *t* test analysis. *p < 0.001. (**E**) HEK293T cells were cotransfected with RORα2 and LSD1 and treated with or without pargyline (3 mM). Pargyline treatment attenuates transcriptional activation of the *ROR*α*2E* promoter-luciferase reporter by LSD1. Cells were transfected with either 100 ng of *RORa2E* luciferase reporter along with 100 ng of RORα2 or 100/400 ng of LSD1. Results are expressed as fold activation compared to empty vector. Data are represented as mean ± S.E.M. for three independent experiments. *P* value is shown from Student’s *t* test analysis. *p < 0.05. (**F**) ChIP assay on the *ROR*α*2E* promoter-luciferase reporter in HEK293T cells with mock or LSD1 shRNA. Occupancy of the promoter by LSD1, RORα2 and RNA polymerase II was analyzed.

Since LSD1 turned out to be a binding partner of RORα2, we next examined whether LSD1 regulates RORα2-mediated transcriptional activity using RORα2E (RORα2 response element)-containing luciferase reporter in HEK293 cells. Overexpression of wild type (WT) of RORα2 was sufficient to potentiate the RORα2E-containing promoter activity, whereas RORα2 E542K mutant which has a mutation in Activation Function 2 (AF2) region failed to activate RORα2E-luciferase activity (Fig. 2A). Co-expression of LSD1 WT with RORα2 further enhanced transcriptional activity of RORα2, whereas LSD1 K661 A mutant that has impaired enzymatic activity failed to further potentiate RORα2 transcriptional activity. Both RORα2 E542K and LSD1 K661 A mutants were able to interact with LSD1 and RORα2, respectively (Fig. 2B and C). Based upon the findings, our data indicate that LSD1 functions as a coactivator for RORα2-dependent transcription. Consistent with the finding that LSD1 potentiates RORα2 transcriptional activity, knockdown of LSD1 using shRNA markedly decreased RORα2E-luciferase reporter activity (Fig. 2D).

To examine whether LSD1 activity is critical for RORα2-dependent transcriptional activity, HEK293 cells were treated with pargyline, an inhibitor of amine oxidase to inhibit LSD1 enzymatic activity and measured the luciferase activity driven by RORα2E-luciferase reporter. Treatment of pargyline significantly diminished RORα2-mediated transcriptional activity (Fig. 2E), indicating that LSD1 enzymatic activity is important for RORα2-dependent transcriptional activity. Further, chromatin immunoprecipitation (ChIP) assay was conducted to examine the recruitment of RNA polymerase II and various histone marks including H3K4me2 and H3K9me2 to the RORα2E-containing promoter in the presence or absence of LSD1. ChIP assay revealed that RNA polymerase II was recruited to the RORα2E-containing promoter concomitant with the recruitment of LSD1 (Fig. 2F). However, knockdown of LSD1 significantly diminished the recruitment of RNA polymerase II, indicating that LSD1 is responsible for transcriptional activation for the RORα2-mediated transcription (Fig. 2F). Indeed, knockdown of LSD1 decreased H3K4me2 but increased H3K9me2 histone mark, confirming that LSD1 functions as a coactivator for RORα2.

### Identification of *CTNND1* as a novel RORα2 target gene that is activated by LSD1

**Figure 3:**
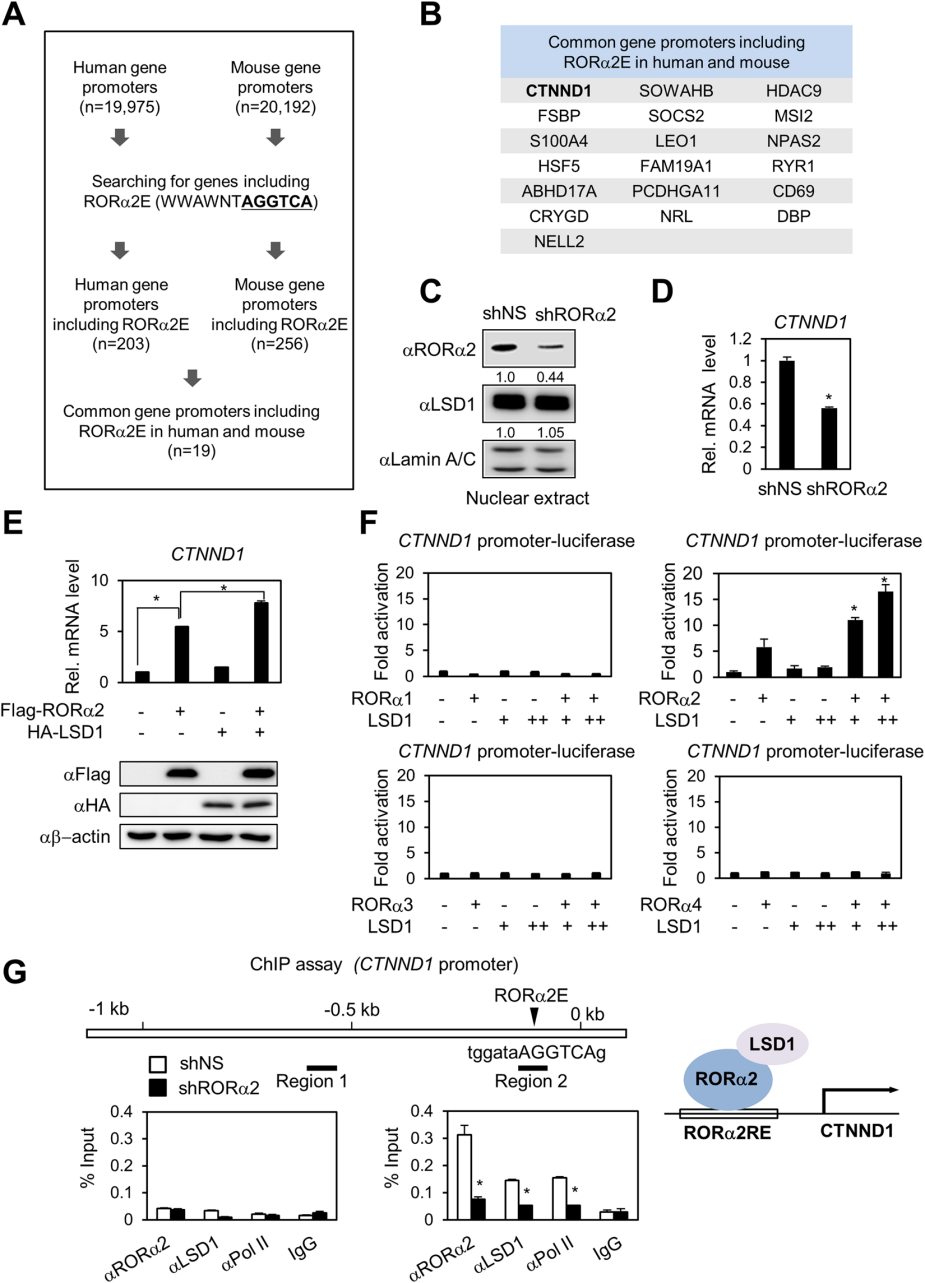
Identification of RORα2-dependent target genes exemplified by *CTNND1*. (**A**) Flow chart showing the strategy for the identification of *ROR*α*2*-dependent genes. W: A, G; N: A, T, G, C. (**B**) Common gene promoters including RORα2 response element (RORα2E) in human and mouse. (**C**) Efficacy of RORα2 knockdown by shRORα2 in HEK293T cells is shown by immunoblot analysis. Band intensities were quantified using Image J, normalized relative to the quantity of their respective anti-Lamin A/C bands. (**D**) Quantitative RT-PCR analysis shows *CTNND1* mRNA level after knockdown of RORα2 in HEK293T cells. The quantity of mRNA was normalized by HPRT. Data are represented as mean ± S.E.M. for three independent experiments. *P* value is shown from Student’s *t* test analysis. *p < 0.001. (**E**) Quantitative RT-PCR analysis of *CTNND1* mRNA level in HEK293T cells in the presence of RORα2 and LSD1. The quantity of mRNA was normalized by HPRT. Data are represented as mean ± S.E.M. for three independent experiments. *P* value is shown from Student’s *t* test analysis. *p < 0.005. Expression of RORa2 and LSD1 was presented by immunoblot analysis (bottom panel). (**F**) Transcriptional activation of the *CTNND1* promoter-luciferase reporter by all RORα isoforms was analyzed. Transcriptional activation of *CTNND1* by RORα2 is enhanced by LSD1 in a dose-dependent manner. Data are represented as mean ± S.E.M. for three independent experiments. *P* value is shown from Student’s t test analysis. *p < 0.001 compared to lane 2. (**G**) ChIP assay was performed on the *CTNND1* promoter in the absence or presence of RORα2 shRNA (left panel). Occupancy of the promoter by LSD1, RORα2, and RNA polymerase II is indicated. Recruitment of LSD1 and RNA polymerase II on RORα2E on the *CTNND1* promoter was restricted by RORα2 knockdown. *P* value is shown from Student’s *t* test analysis. *p < 0.001 compared to control. Upper illustration represents location of RORα2E on *CTNND1* promoter. Proposed model of LSD1 serving as a co-activator for RORα2 transcriptional activity on promoter of target gene, CTNND1 (right panel).

Both RORα1 and RORα2 have been reported to bind DNA as a monomer or homodimers to RORE. Although RORα1 and RORα2 share identical core sequence for RORE, difference in upstream region of 6 bp A/T-rich sequences allows both RORα1 and RORα2 to bind to the promoters of their distinct own target genes^3,22^. Given that WWAWNTAGGTCA is a specific sequence for RORα2E, we performed a search for target gene promoters harboring WWAWNTAGGTCA from the whole genome of human and mouse to identify RORα2-dependent target genes. Nineteen genes were found to be the common genes harboring RORα2E from the both species (Fig. 3A,B and Supplementary Table S1).

RORα2-dependent target genes were selected and validated using shRNA against RORα2. The efficacy of shRNA against RORα2 to reduce RORα2 protein level was validated by immunoblot analysis (Fig. 3C). Indeed, knockdown of RORα2 reduced *CTNND1* mRNA level (Fig. 3D) that was predicted as an RORα2 target gene from the promoter search program for the RORα2 binding site (Fig. 3B). To determine whether LSD1 functions as a coactivator for *RORα*2 target genes, we ectopically overexpressed RORα2 and/or LSD1 in HEK293 cells and examined the mRNA level of *CTNND1*. As RORα2 overexpression increased the mRNA level of *CTNND1* (Fig. 3E), we expected that co-expression of RORα2 and LSD1 further increase *CTNND1* transcription. Co-expression of RORα2 and LSD1 further increased mRNA level of *CTNND1* (Fig. 3E), indicating that LSD1 functions as a coactivator to potentiate *RORα*2 target gene expression. To confirm that *CTNND1* is regulated by RORα2 at transcription level, luciferase reporter assay was performed using *CTNND1* gene promoter-luciferase. While other RORα isoforms barely activated, RORα2 activated *CTNND1* promoter-luciferase activity and LSD1 further increased RORα2-dependent activation in dose-dependent manner (Fig. 3F).

To examine whether RORα2 and LSD1 are co-recruited on *CTNND1* promoter, ChIP assay was performed in the absence or presence of shRNA against RORα2. Both RORα2 and LSD1 were recruited to *CTNND1* promoter along with RNA polymerase II (Fig. 3G). However, knockdown of RORα2 almost completely abolished the recruitment of LSD1 and RNA polymerase II to the *CTNND1* promoter (Fig. 3G), indicating that recruitment of LSD1 to the *CTNND1* promoter is mediated by RORα2. Negative control region which contains no functional RORα2E failed to recruit RORα2 and LSD1. Our data indicate that LSD1 serves as a co-activator for RORα2.

### RORα2 increases cell migration in breast cancer cells

The roles of CTNND1 are controversial in terms of cell adhesive activity; it can positively and/or negatively regulate cell adhesive activity^23^. Alteration of CTNND1 localization or CTNND1 isoform switch has been shown to induce cell migration and invasion^24,25^. CTNND1 promotes Her2/ErbB2-induced breast cancer cell migration and invasion by activating Ras-related C3 botulinum toxin substrate 1 (Rac1) and Cell division cycle 42 (Cdc42)^26^. Furthermore, the binding of CTNND1 to mesenchymal cadherins was required for cell migration and invasion through activation Rac1 in MDA-MB-231 cell lines^27^. Consequently, CTNND1 possesses oncogenic potential depending on its localization and isoform.

**Figure 4:**
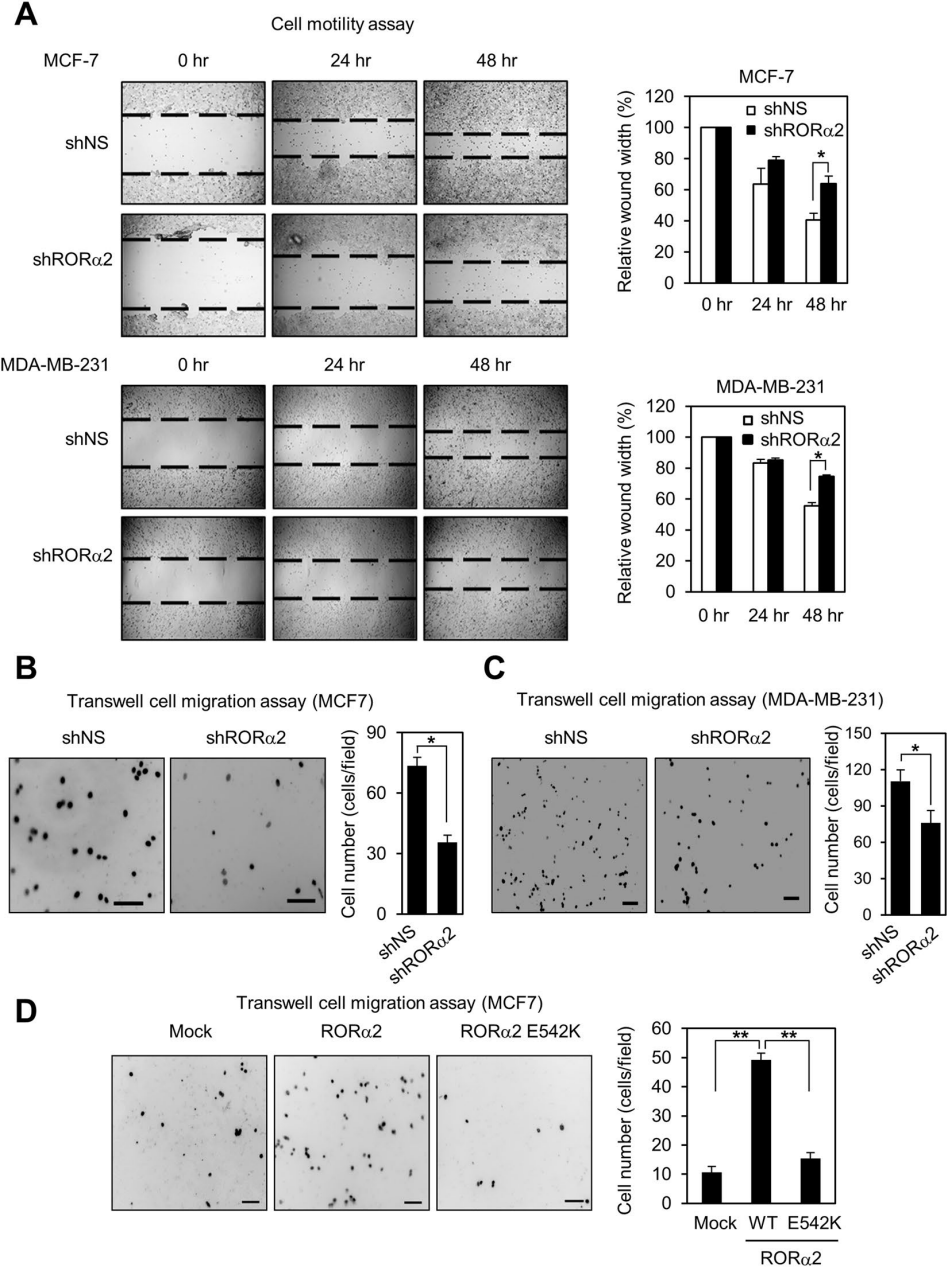
RORα2 increases cell motility and migration in breast cancer cells. (**A**) Photomicrographs from the scratch-motility assay of MCF7 (upper panel) and MDA-MB-231 (bottom panel) cells expressing shRNA against RORα2. MCF7 and MDA-MB-231 cells were wounded with the micro-pipette tip. Wound closure was monitored by photomicrography every 24 hr intervals for 48 hrs. Cell migration (%) was quantified by calculating the wound width as shown in the right panel graph. *P* value is shown from Student’s *t* test analysis. Error bars represent S.E.M. *p < 0.005. (**B–D**) Photomicrographs (100X) from transwell migration assay of MCF7 cells (**B**) and MDA-MB-231 cells (**C**) expressing shRNA against RORα2 or MCF7 cells expressing shRNA against RORα2 WT or RORα2 E542K (**D**) for 16 hrs. The bar in photomicrographs represents 100 μm. Graphs show the mean number of migrated cells per field, and *p* value is shown from Student’s *t* test analysis. Error bars represent S.E.M. *p < 0.005; **p < 0.001.

Given that RORα2 activates gene expression of CTNND1, we examined if RORα2 affects cell motility or invasiveness mediated by CTNND1. Knockdown of RORα2 largely reduced cell motility in breast cancer cells, including MCF7 cells and highly metastatic MDA-MB-231 cells (Fig. 4A). Similarly, knockdown of RORα2 largely reduced cell migration in MCF7 cells and MDA-MB-231 cells (Fig. 4B and C). Consistently, RORα2 overexpression markedly increased cell migration, whereas RORα2 E542K mutant reduced cell migration in MCF7 cells (Fig. 4D). Together, our data indicate that RORα2-mediated transcriptional activation positively regulates cell motility and migration in breast cancer cells.

### RORα2 and LSD1 protein levels are markedly elevated in human breast cancer

**Figure 5:**
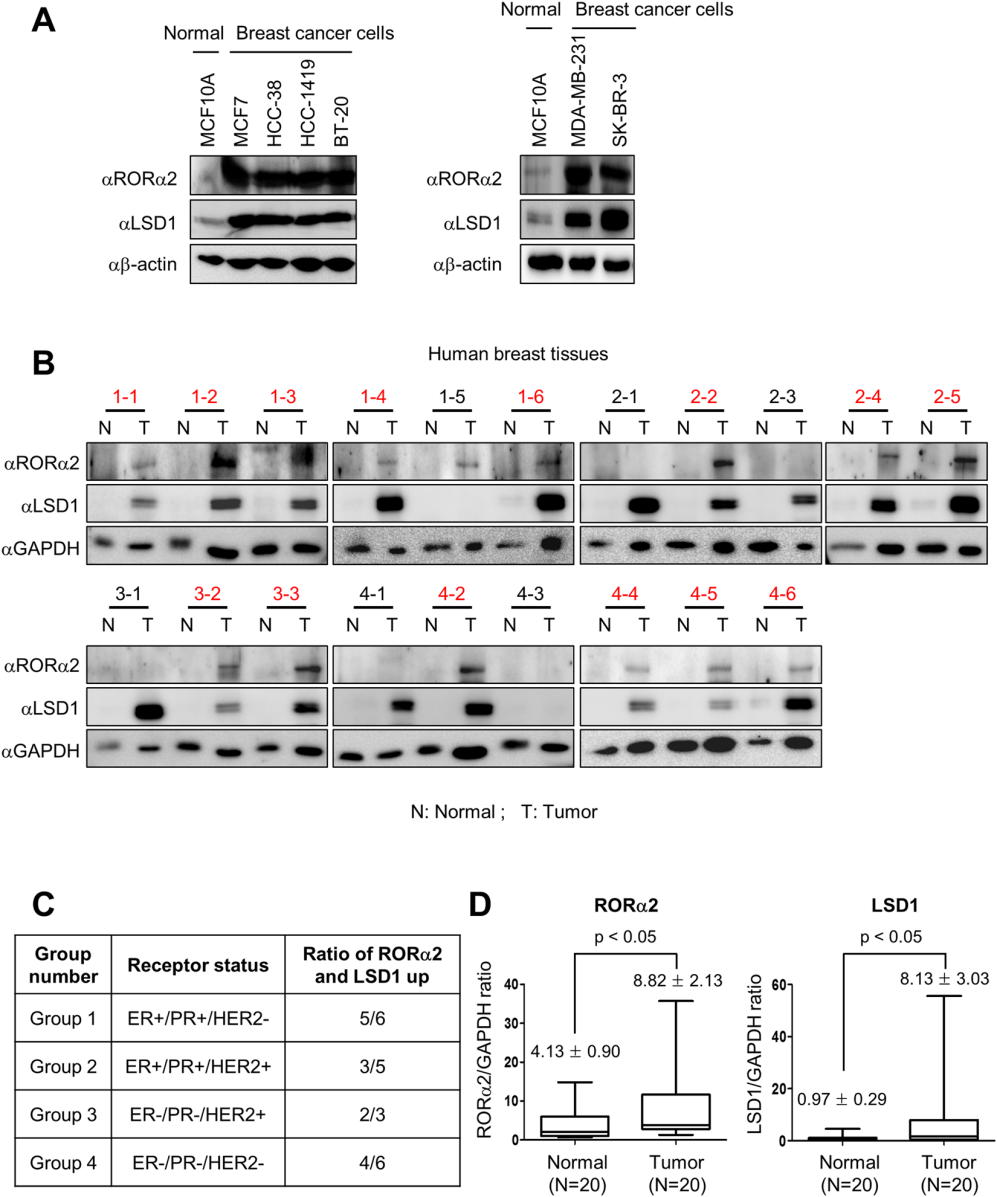
Increased RORα2 and LSD1 expression in human breast tumor tissues. (**A**) Protein levels of RORα2 and LSD1 in normal and breast cancer cell lines. (**B**) Protein levels of RORα2 and LSD1 in 20 human breast tumor tissue samples (T) along with matching normal tissue samples (N). The red character means a patient sample with both RORα2 and LSD1 protein levels increased. (**C**) Table shows the ratio of patients with increased protein levels of both RORα2 and LSD1 by breast cancer types. (**D**) Statistical analysis of the ratio of RORα2 (left panel) and LSD1 (right panel) to GAPDH in 20 human breast tumor samples compared to normal specimen calculated using paired *t*-test. Data are presented as bar and whisker graphs, showing the median and the distribution of 50% (bar) and 99% (whisker) of all specimen examined.

We have reported that RORα plays critical roles to reduce tumor progression by attenuating WNT/β-catenin signaling and by enhancing p53-dependent apoptotic function^2,19^. To examine the roles of RORα2 in human cancer, we analyzed the protein levels of RORα2 and LSD1 in various breast cancer cell lines. Protein levels of both RORα2 and LSD1 were remarkably increased in various breast cancer cell lines compared to the normal breast cell line (Fig. 5A). To find the clinical relevance of our data that RORα2 and LSD1 protein levels are remarkably increased in breast cancer cell lines, we analyzed the protein levels of RORα2 and LSD1 in human breast cancer specimens along with normal counterparts. Immunoblot analysis revealed the increase of both RORα2 and LSD1 protein levels in human breast cancer samples compared with their normal counterparts (Fig. 5B). The increment of RORα2 and LSD1 protein levels was not dependent on breast cancer type (Fig. 5C). Statistical analysis confirmed the significant elevation of RORα2 and LSD1 protein levels in human breast cancer (Fig. 5D). Taken together, these results indicate the protein levels of RORα2 and LSD1 are elevated in human breast cancers compare to their normal counterparts.

## Discussion

Numerous studies have provided evidence that RORα plays a role in cancer. The *Rorα* gene is located in chromosomal band 15q22.2 harboring the common fragile site FRA15A^28^. Thus, location of the *Rorα* gene in common fragile site FRA15A has implicated genetic alteration and genomic instability of *Rorα* gene. The observation that *Rorα* mRNA level is often down-regulated in cancer cell lines and human cancer tissues^28,29^ support the potential tumor suppressive function of RORα. Furthermore, gene expression profile studies in various cancers have shown that *Rorα* is a common down-regulated gene in certain cancer types, including breast cancer^29^. Consistent with previous reports, we have reported that RORα attenuates Wnt/β-catenin signaling by PKCα-dependent phosphorylation in colon cancer cells and enhances p53-dependent apoptotic function to attenuate tumor progression^2,19^. In addition, synthetic RORα agonist has been shown to induce apoptosis by p53 stabilization, implicating a potential to be developed for the therapeutic reagent of human cancer^30^.

Here, we report that RORα2 plays a critical role to promote cell migration and motility in human breast cancer cells. We searched for RORα2-dependent target gene promoters containing RORα2E. Using bioinformatics tools, we identified a putative *RORα*2 target gene, *CTNND1* that has been known as a critical component of the adherent junction and a potential activator of Rac1, Cdc42, and ras homolog gene family member A (RhoA)^31^. Although CTNND1 has been reported to suppress cell migration and invasion^25,32^, alteration of CTNND1 localization can promote cell migration and invasion. If protein level of CTNND1 is increased, RhoA activity is reduced whereas Rac1 and Cdc42 activity is elevated to promote cell migration^33^. Alteration of CTNND1 localization was observed to promote tumor progression in human breast cancer^34^. Our studies demonstrate that RORα2 positively regulates CTNND1 expression to increase cell motility and migration in breast cancer cells. These results implicate that the oncogenic function of CTNND1 might be, at least in part, regulated by RORα2 transcriptional activity.

Oncogenic roles of LSD1 in various types of cancers have been widely reported. Overexpression of LSD1 in prostate cancer is sufficient to promote androgen receptor-dependent transcription in the absence of androgens^17,35^. In addition, LSD1 has been shown to participate in maintaining the undifferentiated and malignant phenotype of neuroblastoma cells^36^ and the protein level of LSD1 is highly elevated in ER-negative breast cancers^16^. Though it is still probable that RORα2 may form various transcriptional coactivator complexes to activate its target gene expressions, LSD1 plays as a critical transcriptional coactivator to promote cell motility and migration via potentiating RORα2 transcriptional activity in breast cancer.

Our findings demonstrate a clinical relevance that the protein levels of RORα2 and LSD1 are highly elevated in breast cancer specimens compared with their normal counterparts. Especially, we observed that protein levels of RORα2 and LSD1 were largely increased in four types of receptor status in breast cancer specimens. These data strongly suggest that RORα2 and LSD1 may be novel therapeutic targets for human breast cancer. While retaining all beneficial features of RORα in breast cancer cells, our results propose that RORα2 and LSD1 may play crucial roles in tumorigenesis via elevating CTNND1 expression in human breast cancer. The therapeutic approaches to selectively target RORα2 transcriptional activity may provide additional therapeutic strategies to treat human breast cancer.

## Methods

### Antibodies

The following commercially available antibodies were used: anti-Flag (Sigma), anti-HA (Roche), anti-LSD1 (Cell signaling), anti-RNA Polymerase II (Berkeley Antibody Company), anti-β-actin (Santa Cruz), anti-GFP (Santa Cruz), anti-Lamin A/C (Santa Cruz) and anti-GAPDH (Santa Cruz). Anti-RORα2 antibody (target epitope is GKPPYSQKEDKEVQT-C) was generated by Abmart (China).

### Purification and Identification of Binding Proteins for RORα2

RORα2-binding proteins were affinity-purified from extracts of HEK293 cells stably expressing Flag-tagged RORα2. The RORα2-binding proteins were immunoprecipitated using anti-Flag antibody-conjugated agarose beads (80 μl of 50% slurry) from about 90 mg of extracts that were washed with buffer containing 20 mM Tris-HCl (pH 7.9), 15% Glycerol, 1 mM EDTA, 1 mM dithiothreitol (DTT), 0.2 mM PMSF, 0.05% Nonidet P40, and 150 mM KCl to remove non-specific contaminants, and the bound materials were eluted by competition with the Flag peptide (0.1 mg/ml). The bound proteins were resolved by sodium dodecyl sulphate-polyacrylamide gel electrophoresis (SDS-PAGE) and prepared for LCMS/MS analysis.

### LC-MS/MS and SEQUEST Analyses

Peptide samples were injected to a column by a Surveyor autosampler (Surveyor, Thermo Finnigan, San Jose, CA) and separated by C18 column. The eluent was directly transferred to the electrospray ionization source of a Thermo Finnigan LCQ DecaXPplus ion trap mass spectrometer. Automated peak recognition, dynamic exclusion, and daughter ion scanning of the two most intense ions were performed and analyzed by the XCALIBUR software. The SEQUEST algorithm was used to interpret MS/MS.

### Luciferase Reporter assays

HEK293T cells were grown and transiently transfected by using Polyethylenimine (PEI). For luciferase reporter assays, 1 × 10^5^ cells were seeded in DMEM supplemented with 5% FBS for 24 hr. Cells were transfected with either 100 ng of *RORa2E* or *CTNND1* promoter reporter along with 100 ng of RORα2 or 100/400 ng of LSD1. Using a luciferase assay system (Promega), the luciferase activity was measured using a luminometer 48 hr after transfection and transfection efficiency was normalized by β-galactosidase expression. Values are expressed as means ± S.E.M. for at least three independent experiments.

### Chromatin Immunoprecipitation (ChIP) assays

The ChIP assay was conducted using sheared genomic DNA fragments with an average fragment size of approximately 300 bp to 1 kb. Eluted components were diluted 1:10 with ChIP dilution buffer (20 mMol Tris-HCl (pH 8.1), 150 mMol NaCl, 2 mMol ethylenediaminetetraacetic acid (EDTA), and 1% Triton X-100). For PCR, 1 μl from 30 μl DNA extract and 25-30 cycles of amplification were used. For the shRNA-coupled ChIP assay, MCF7 cells expressing each shRNA were harvested and the immunoprecipitated chromatin was analyzed by PCR with primers specific to the promoters. The following primers were used: RORα2E promoter-luciferase sense strand 5′-CGGTACTGTTGGTAAAATGG-3′ and antisense strand 5′-GCAATTGTTCCAGGA ACCAG-3′; *CTNND1* promoter PCR2 (containing RORα2E) sense strand 5′-CCCTGTCTTTCTCTCCTCTCTTTTT-3′ and antisense strand 5′-AAGTGATGTCAGCCCCTGTGA-3′; CTNND1 promoter PCR1 (negative region) sense strand 5′-TCAGGGAAAAATAATCCAATCTCAT-3′ and antisense strand 5′-GCTTTCTTCAACATCCCACCAG-3′.

### RNA Preparation and Reverse Transcription PCR (RT-PCR)

Total RNA was isolated from the cells using TRIZOL reagent (Invitrogen, Grand Island, NY) according to the manufacturer’s instructions. First strand cDNA was synthesized with 2.5 μg of each of the RNA samples primed with random hexamers via M-MLV reverse transcriptase (Fermentas, Burlington, Canada), and synthesized cDNA was then amplified by real-time quantitative RT-PCR.

### Real-Time Quantitative RT-PCR

The mRNA was detected by an ABI prism 7300 system with SYBR Green (molecular probes). Primer pairs were designed to amplify 90–150 bp mRNA specific fragments, and confirmed to be unique products by melting curve analysis. The PCR conditions were 95 °C (5 min) and 40 cycles of 95 °C (30 sec), 57 °C (30 sec), and 72 °C (30 sec). The quantity of mRNA was calculated using the ΔΔCt method and normalized by using primers to detect HPRT. All reactions were performed in triplicates. The following primers were used: hCTNND1, 5′-CCGGGTCTCACCACAAGATG-3′ and 5′-GGGGTCCGTTGAGTTTCAAAT-3′; hLSD1, 5′- GATCTGACCGCCCTATGCAA-3′ and 5′- AGTTGAGAGAGGTGTGGCATTAGC-3′, hHPRT, 5′-TGACACTGGCAAAACAATGCA-3′ and 5′-GGTCCTTTTCACCAGCAAGCT-3′.

### *In Vitro* Cell Motility Assay

To analyze the two-dimensional motility, a wound-healing scratching motility assay was performed. MCF7 and MDA-MB-231 cells that transient transfected with shNS or shRORα2 were seeded in 6-well culture plates and cultured until they reached confluence. The cells were scratched with a 200 μl micro-pipette tip. The plates containing these cells incubated at 37 °C for 48 hr. Photomicrographs of the closed gap were captured at 0 hr, 24 hr, and 48 hr of incubation using an EVOS xl transmitted light microscope (AMG, Bothell, WA). Migration distance of the cells was quantified by distance of gap. Values are expressed as means ± S.E.M. for three independent experiments.

### Transwell Cell Migration Assay

MCF7 cells transiently transfected RORα2, RORα2 E542K, or RORα2 shRNA and MDA-MB-231 cells transiently transfected RORα2 shRNA were used in Transwell cell migration assays. Transwell cell migration assay was conducted as previously described^2^. 1 × 10^4^ MCF7 cells and MDA-MB-231 cells were loaded onto the top of a 24-well Transwell chamber assay plate (BD Biocoat, BD Biosciences). Conditioned DMEM medium containing 10% fetal bovine serum was added to the bottom chamber as a chemoattractant. After 16 hr incubation, the cells that had migrated to the lower chamber of the filter were fixed with 100% methanol, stained with DAPI, and quantified by counting the total number of cells in five different fields. All experimental studies were performed according to the manufacturer’s protocols. Values are expressed as means ± S.E.M. for three independent experiments.

### Human Breast Cancer Tissue Specimens

For the analysis of RORα2 and LSD1 expression in human tissue samples, 20 paired fresh frozen breast cancer tissues and matched normal tissues were selected. The methods were carried out *in accordance with* the relevant guidelines and regulations. The informed consents to use the tissue specimens for research purposes were obtained from patients, and the utilization of the specimens for this research was authorized and approved by the Institutional Review Board of College of Medicine, Seoul National University (1704–015–842). All experiments were carried out in accordance with approved guidelines.

## Electronic supplementary material

Supplementary Information Guide

Supplementary Table S1
