# Supplementary material for: RORα2 requires LSD1 to enhance tumor progression in breast cancer
Source: Sci Rep. 2017 Sep 20;7:11994. doi: 10.1038/s41598-017-12344-0 (PMC5607251; doi:10.1038/s41598-017-12344-0)
Supplement: Supplementary file 1 — Supplementary Information Guide [file 41598_2017_12344_MOESM1_ESM.pdf]

**Supplementary Information Guide**

To accompany submitted manuscript entitled: **"RORα2 requires LSD1 to enhance tumor progression in breast cancer"** (Kyeongkyu Kim, Ji Min Lee, Young Suk Yu, Hyunkyung Kim, Hye Jin Nam, Hyeong-Gon Moon, Dong-Young Noh, Keun Il Kim, Sungsoon Fang and Sung Hee Baek)

**Supplementary Figure S1. Full-length blots**

Documents included (Excel file):

**Supplementary Table S1. List of human and mouse genes having RORα2 response element on the promoters**

**Supplementary Figure S1. Full-length blots**

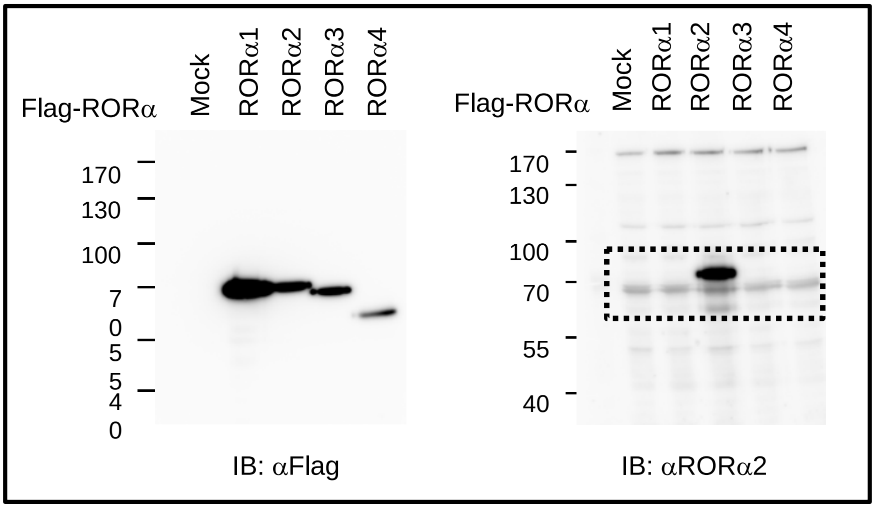


Figure 1C

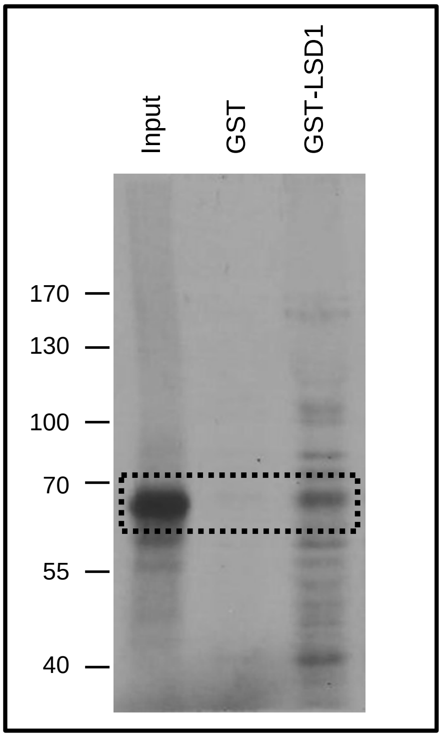


Figure 1E

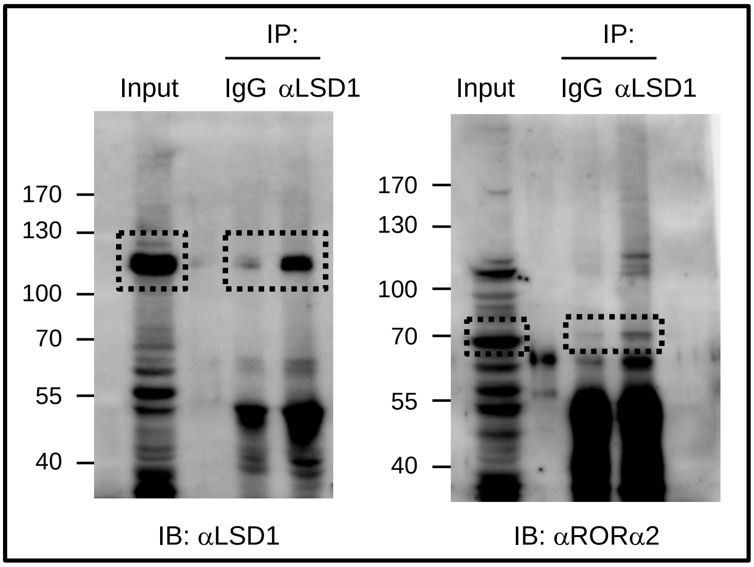


Figure 1D

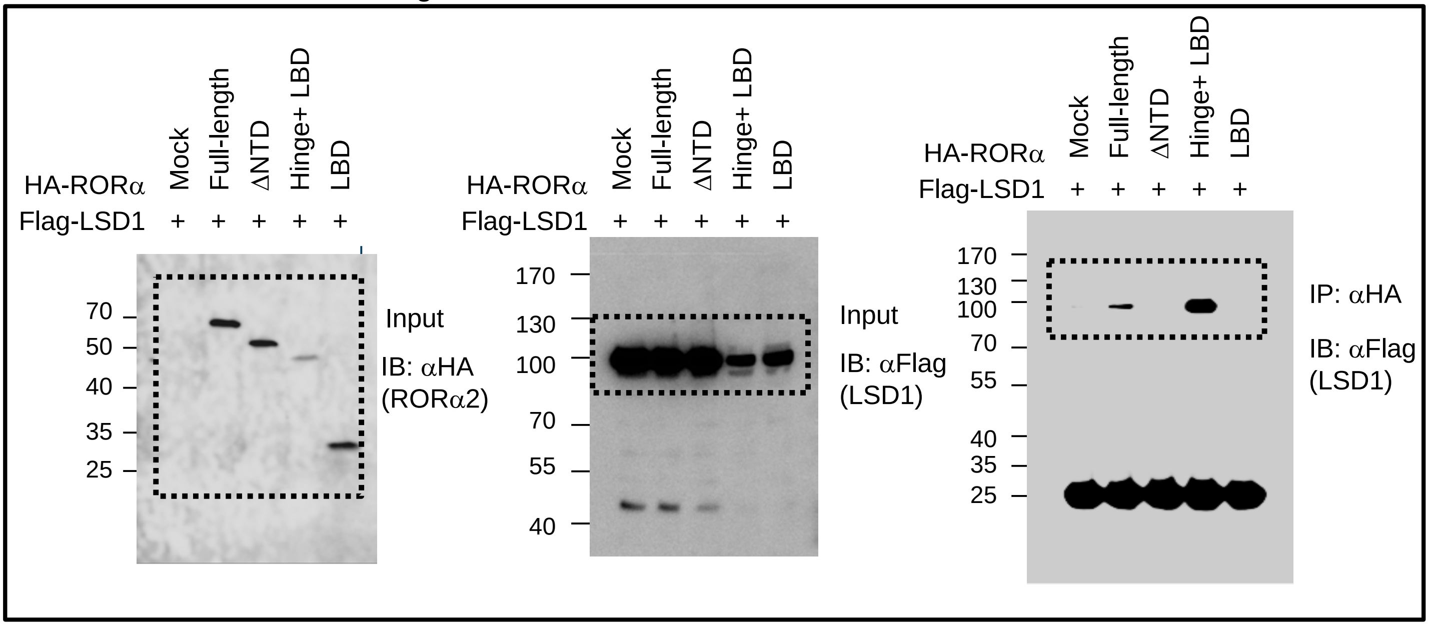


Figure 1F

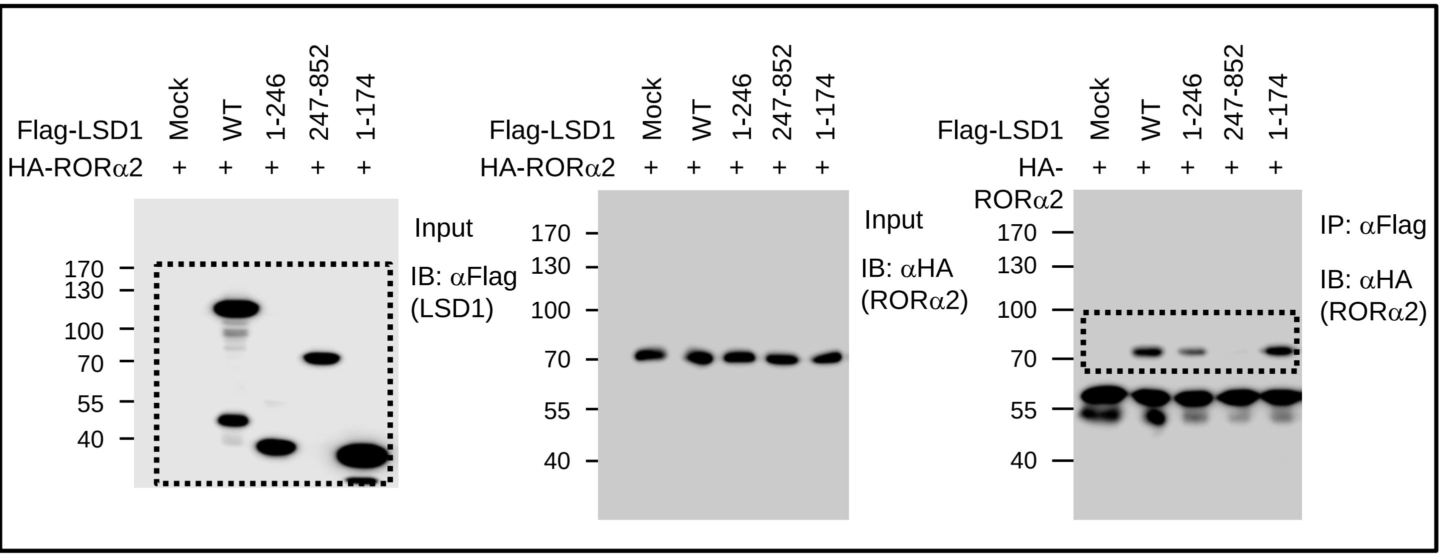


Figure 1G

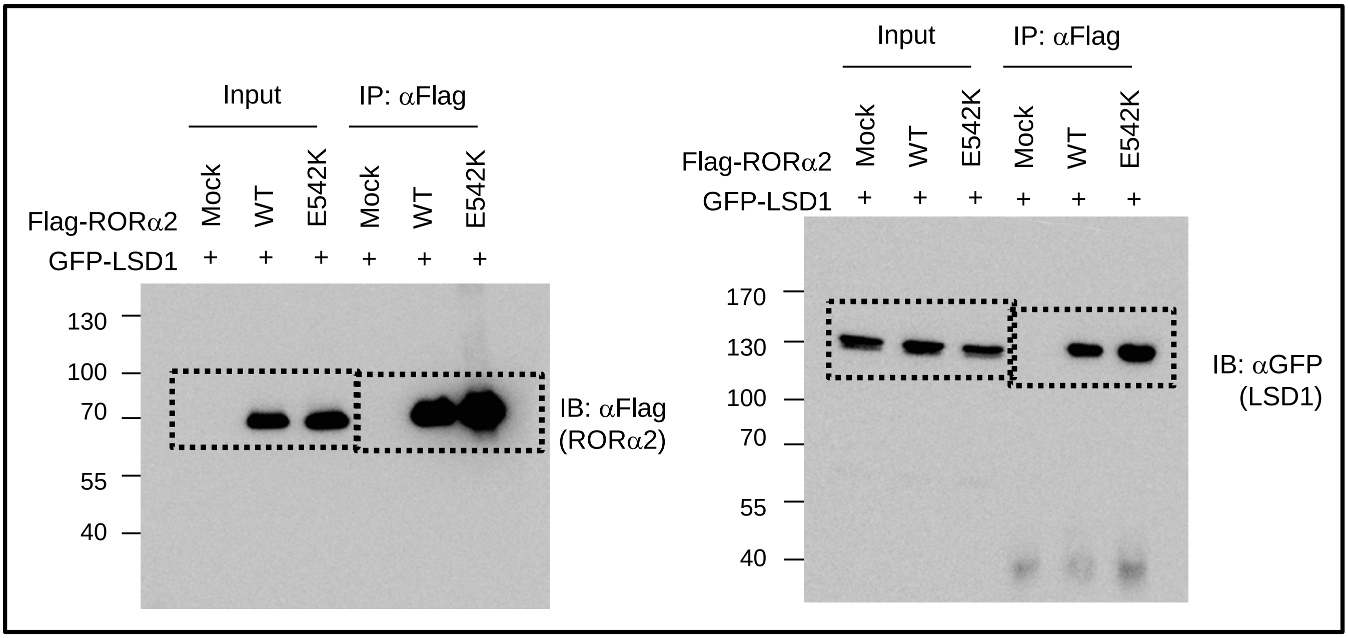


Figure 2B

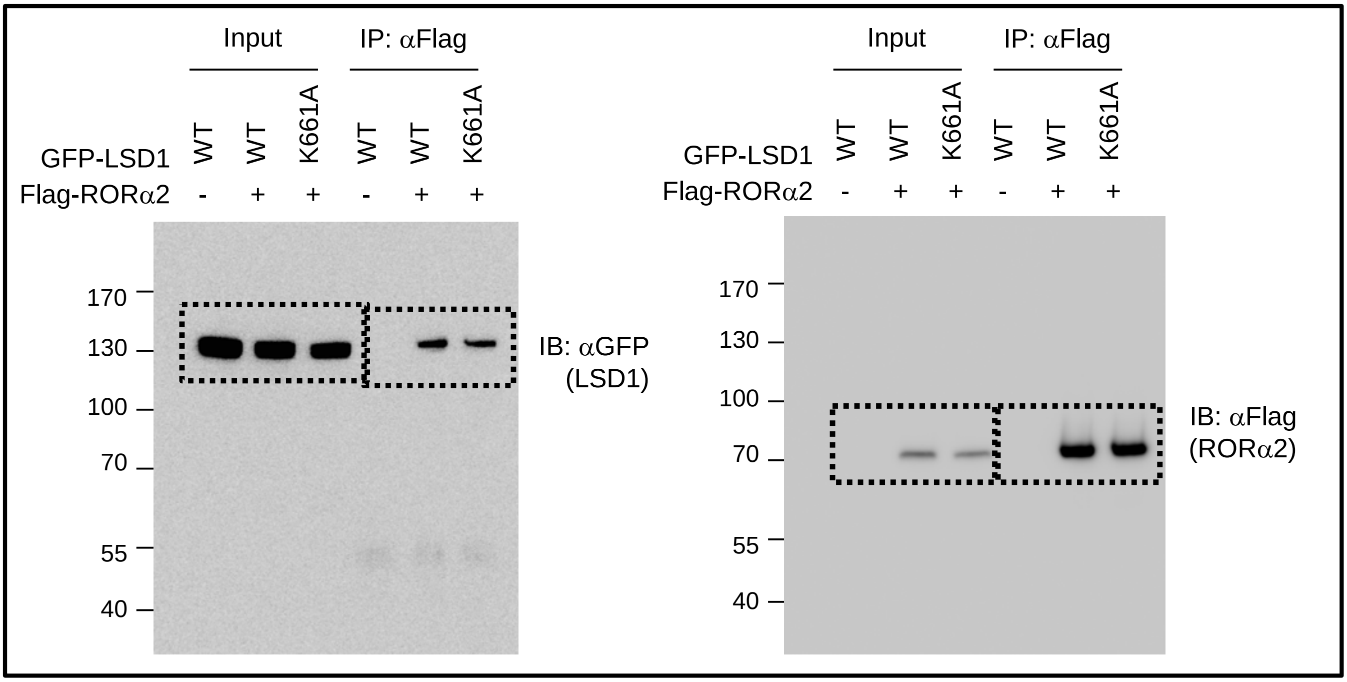


Figure 2C

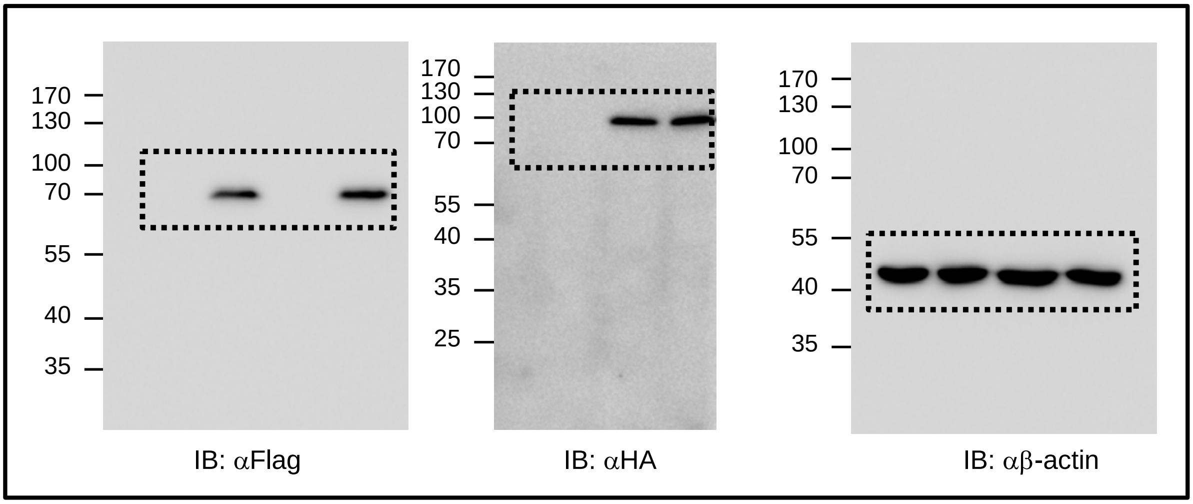


Figure 3E

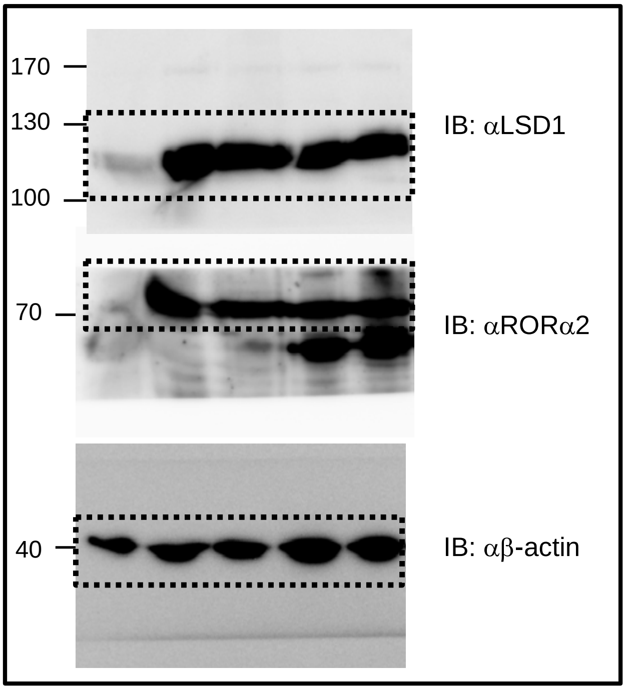


Figure 5A
left pannel

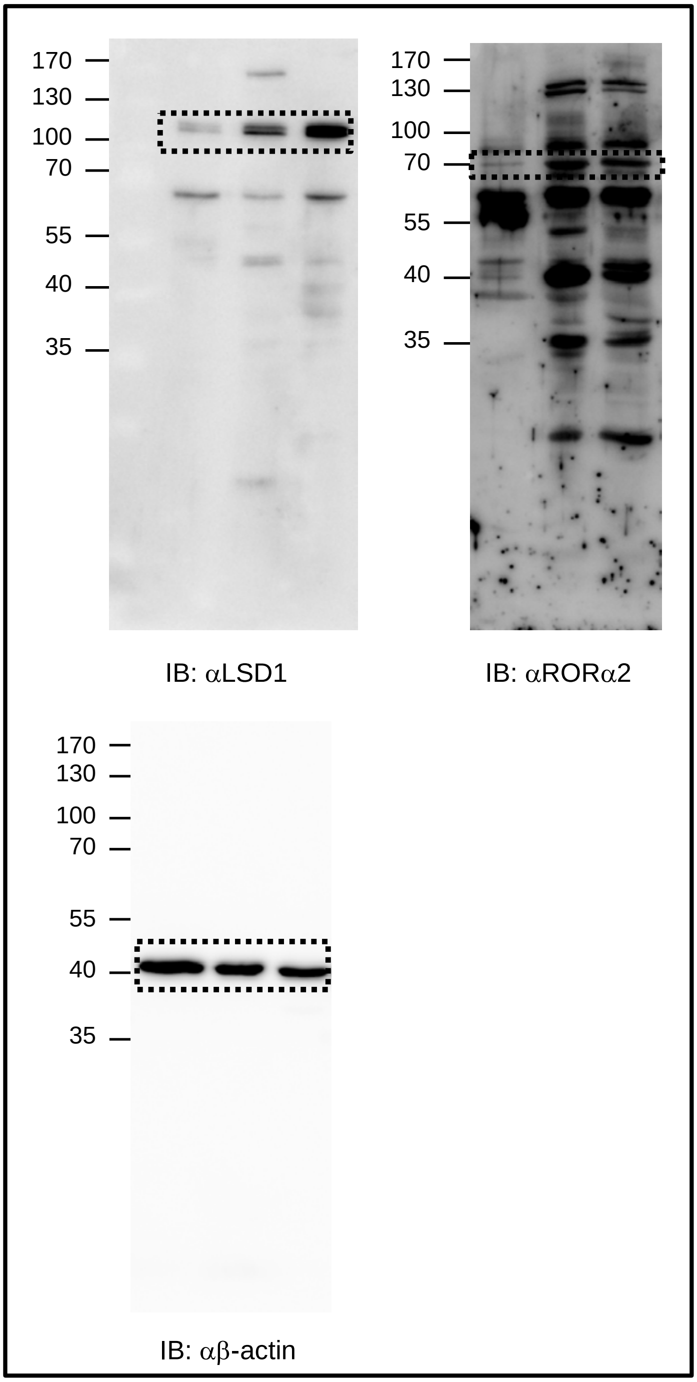


Figure 5A
right pannel

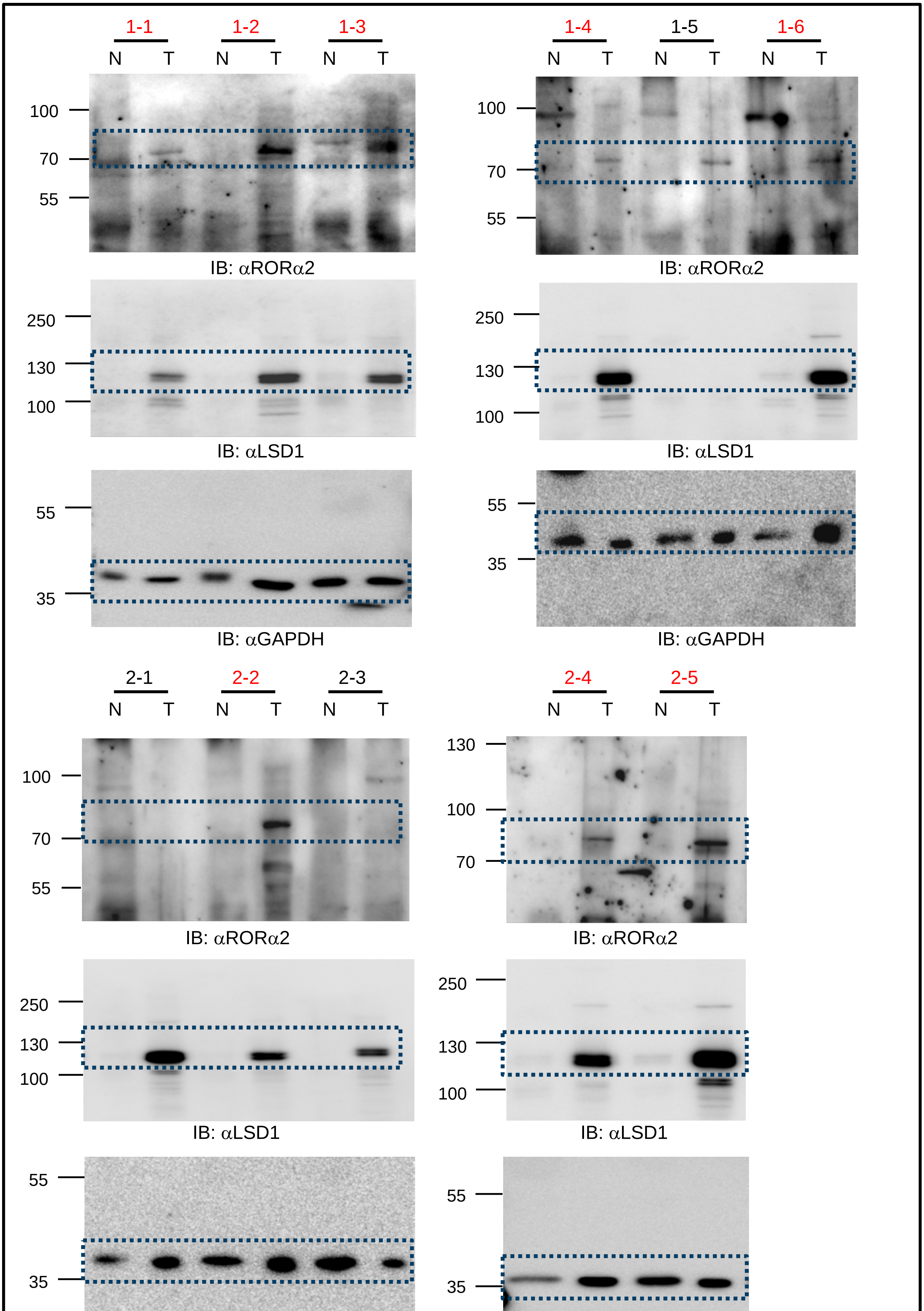
1-1
1-2
1-3
N T N T N T
100
70
55
IB: αRORα2
250
130
100
IB: αLSD1
55
35
IB: αGAPDH
1-4
1-5
1-6
N T N T N T
100
70
55
IB: αRORα2
250
130
100
IB: αLSD1
55
35
IB: αGAPDH
2-1
2-2
2-3
N T N T N T
100
70
55
IB: αRORα2
250
130
100
IB: αLSD1
55
35
IB: αGAPDH
2-4
2-5
N T N T
130
100
70
IB: αRORα2
250
130
100
IB: αLSD1
55
35
IB: αGAPDH

Figure 5B

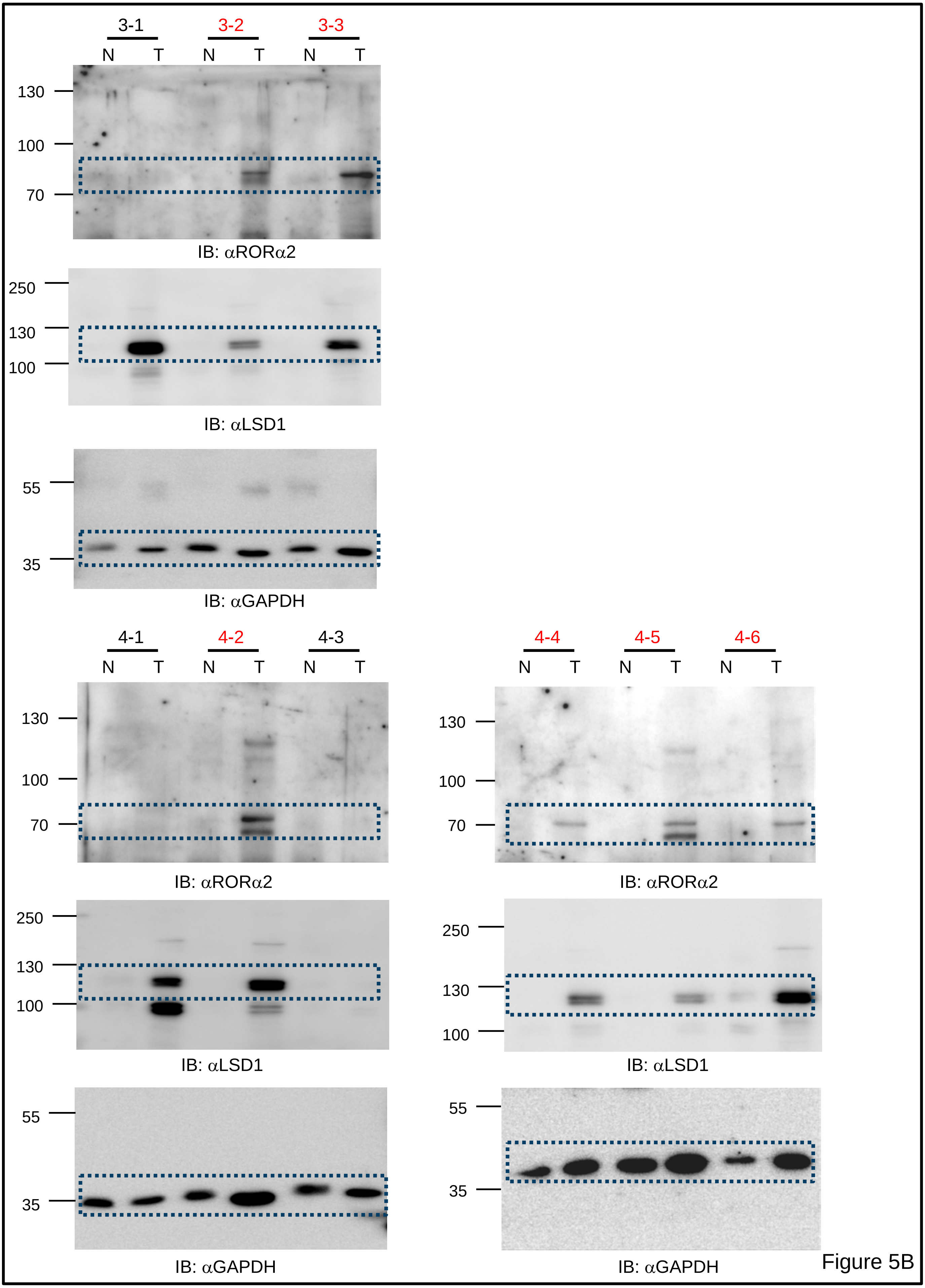
3-1
3-2
3-3
N T N T N T
130
100
70
IB: αRORα2
250
130
100
IB: αLSD1
55
35
IB: αGAPDH
4-1
4-2
4-3
N T N T N T
130
100
70
IB: αRORα2
250
130
100
IB: αLSD1
55
35
IB: αGAPDH
4-4
4-5
4-6
N T N T N T
130
100
70
IB: αRORα2
250
130
100
IB: αLSD1
55
35
IB: αGAPDH

Figure 5B
